# The burden of care: Health and wellbeing of informal caregivers of people with amyotrophic lateral sclerosis

**DOI:** 10.1017/S1478951526101825

**Published:** 2026-02-25

**Authors:** Filipe Gonçalves, Teresa Machado, Pedro Viegas, Ana Machado, Carla Ribeiro

**Affiliations:** 1 Faculty of Health Sciences, University of A Coruña, A Coruña, Spain; 2 APELA – Portuguese Association of Amyotrophic Lateral Sclerosis, Porto, Portugal; 3 Portuguese Oncology Institute of Porto (IPO-Porto)/ Porto Comprehensive Cancer Center (P.CCC), Rise@Ci-POP (Health Research Network), Porto, Portugal; 4 Pulmonology Department, Hospital Santos Silva/Unidade Local de Saúde de Gaia e Espinho, Vila Nova de Gaia, Portugal; 5 MEDCIDS-Department of Community Medicine, Information and Health Decision Sciences, Faculty of Medicine, University of Porto, Portugal; 6 CINTESIS@Rise-Health, Faculty of Medicine, University of Porto, Porto, Portugal

**Keywords:** Caregivers, amyotrophic lateral sclerosis, caregiver burden, health-related quality of life, mental health

## Abstract

**Objectives:**

Amyotrophic lateral sclerosis (ALS) is a rare, progressive, and fatal disease that impacts the lives of affected individuals and their caregivers. Informal caregivers play a crucial role in supporting people with ALS (pwALS), yet they face major challenges. This study aims to analyze caregiver burden and health status among informal caregivers of pwALS in Portugal.

**Methods:**

A cross-sectional survey-based study was conducted with adult informal caregivers of pwALS in Portugal, recruited through the Portuguese ALS patient association and healthcare professionals. Data included sociodemographics, caregiving activities, caregiver health (SF-36), patient functional status (ALSFRS-R), and caregiver burden (ZBI).

**Results:**

The study included 113 caregivers. Most were female (61.9%) and the partner (65.5%) or offspring (23.9%) of the pwALS. A quarter of caregivers received no social benefits. Mean ZBI was 32 ± 14.8, with most reporting mild to moderate burden. On the SF-36, general health was 51.1 ± 19.8, with mental health (55 [40; 70]) and vitality (43.8 [31.3; 56.3]) particularly impaired. ZBI scores correlated positively with caregiving hours (*r* = 0.274, *p* = 0.003) and negatively with ALSFRS-R (*r* = −0.411, *p* < 0.001). High burden caregivers exhibited poorer sleep quality (*p* =  0.026).

**Significance of results:**

Caregivers experienced mild to moderate burden, with impaired mental health and vitality, but preserved physical functioning. A higher burden was linked with lower quality of life, poorer sleep, and greater patient disability. These findings underline the need for targeted education and training to support caregivers of pwALS.

## Introduction

Amyotrophic lateral sclerosis (ALS) is a rare, progressive, and fatal degenerative neurological disease of the motor neurons that leads to progressive paralysis of the limbs, bulbar, and respiratory muscles (Masrori and Van Damme 2020).

Given the complex needs of people with ALS (pwALS), caregivers play a crucial role in the management of the disease. This role is most often taken on by family members or close friends – informal caregivers – who are usually unpaid and face significant emotional, physical, social, and financial challenges (Conroy et al. 2023).

As the disease progresses, the demands on caregivers increase significantly (de Wit et al. 2018), initially through assistance of basic daily tasks, and later with complex care, including the coordination of medical services and rehabilitation routines (Munan et al. 2021; Gonçalves and Magalhães 2022). Advanced roles involve the management of home mechanical ventilation (HMV), for respiratory failure (Kaub-Wittemer et al. 2003), management of in-exsufflator devices in assisted cough, or management of alternative or complementary enteral feeding routes, such as gastrostomy (Conde et al. 2018).

This ever-growing burden, commonly referred to as caregiver burden, impacts the overall health related quality of life (HRQoL) of caregivers, with physical, psychological, and spiritual stress (Brandstötter et al. 2025). In addition to the logistical challenges of caregiving, high levels of stress, anxiety, depression, and fatigue are reported by caregivers of pwALS (Del-Pino-Casado et al. 2021).

Anxiety is a prevalent complaint in the early stages of the disease and emerges as a strong predictor of caregiver burden and a significant risk factor for psychological morbidity (Burke et al. 2018; Larsson et al. 2023). Throughout the journey of care, caregivers’ quality of life often deteriorates with increasing responsibilities, further declining their physical health and emotional well-being (Ipek and Güneş Gencer 2024).

Social isolation is an additional commonly reported problem, due to the time-consuming and restrictive nature of caring (Tülek et al. 2023). Additional financial hardships may arise from accumulating medical expenses, necessary home modifications, and lost income as care needs increase (Gladman et al. 2014; Nivakoski and Baggio 2025).

Caregivers of pwALS experience high levels of psychological distress, influenced by factors such as the patient’s functional status, the caregiver’s psychological resilience, and the lack of social support. These concerns are frequently underaddressed, and national data is lacking. Our study aims to ascertain the caregiver burden among informal caregivers of pwALS in Portugal.

## Methods

### Study design

A cross-sectional, web-based survey was conducted in Portugal. The survey link was disseminated nationwide to informal caregivers of pwALS through the Portuguese ALS patient association’s members email and newsletter, and via the HMV Assembly of the Portuguese Respiratory Society, which invited physicians to share it with their patients and caregivers. Data collection was carried out between March 2024 and April 2025.

### Participants

Participants were recruited sequentially via email invitations from clinicians at a healthcare facility. Inclusion criteria were participants aged 18 years or older, who were caring for a pwALS, who were informal caregivers, and who consented to the study. Exclusion criteria were individuals who were not the primary caregiver and caregivers who were unable to communicate effectively in the study language (Portuguese). We powered the study to estimate the mean caregiver burden, Zarit Burden Interview (ZBI), with a 95% confidence interval width corresponding to a ±3-point margin. Assuming a standard deviation of 15 from previous studies, the required sample size was 96 participants. Allowing for 15% missing/incomplete responses, 113 informal caregivers were targeted (Tang et al. 2021; Kennedy et al. 2022; Tülek et al. 2023).

### Measures

Sociodemographic characteristics (age, sex, employment status), caregivers reported comorbidities, subjective caregivers’ sleep quality, time spent caring, formation/training received, and list of activities for which the pwALS needed help (i.e., putting on and removing the ventilator mask, performing the in-exsufflator, toileting, preparing food, feeding, getting in/out of bed, sitting/rising, using electronic devices, bathing, dressing/undressing, moving around and leaving the house) were assessed.

Sleep quality was assessed using a subjective 5-point scale, where 1 corresponded to “very poor,” 2 to “poor,” 3 to “fair,” 4 to “good,” and 5 to “very good.” Caregivers were asked to rate both their current sleep quality and their sleep quality prior to assuming caregiving responsibilities.

The Amyotrophic Lateral Sclerosis Functional Rating Scale-Revised (ALSFRS-R) was used to assess the functional status of the pwALS. This scale ranges from 0 to 48 points, with higher scores indicating better physical function and prognosis. ALSFRS-R scores have been associated with higher ZBI scores, reflecting increased caregiver burden (Burke et al. 2018; Bakker et al. 2020).

The HRQoL of caregivers was assessed using the Short Form-36 (SF-36v2) Health Status Questionnaire, with a scale of 0 to 100 points, with higher scores indicating better health status. Caregiver burden was assessed using the ZBI, with a total score ranging from 0 to 88 (≤21: no overload; 21–40: moderate overload; 41–60: moderate to severe overload; ≥61: severe overload). The ZBI has demonstrated that higher caregiver burden correlates with lower quality of life scores (Kühnel et al. 2020; Seyyah et al. 2025). The ALSFRS-R, SF-36, and ZBI are all suitable for self-use in online settings and maintain proper validity when compared to in-person evaluations (White et al. 2018; Bakker et al. 2020; Kühnel et al. 2020).

To minimize measurement bias, standardized instructions and structured, closed-ended questions were used throughout the questionnaire, and data were reviewed for completeness and internal consistency prior to analysis.

### Data analysis

Data were analyzed using SPSS Statistics, version 29.0.0 (IBM Corp., Armonk, NY, USA). The level of significance was set at 0.05. Categorical variables were expressed as frequencies and percentages, and continuous variables were expressed as mean ± standard deviation or median (interquartile range), as appropriate.

Descriptive statistics were used to describe the sample, and the normality of the data was explored with the Kolmogorov–Smirnov test.

We categorized caregivers according to burden level, using a mean ZBI score of ≥24 to define “high burden” (Burke et al. 2018). Differences between participants with low and high burden were explored using independent *t*-tests, Mann–Whitney *U* tests, and chi-squared tests, as appropriate. To adjust for potential confounding factors – namely, caregivers’ age, sex, socioeconomic status, and previous caregiving experience – variables related with caregiver burden, quality of life, and sleep were explored with analysis of covariance.

Correlations between ZBI scores and the other outcomes were explored using Pearson and Spearman correlation coefficients, accordingly.

## Results

In the caregiver survey, 114 responses were received, of which 112 were complete and 2 were partial. Of the partial responses, 1 completed only the informed consent page and was therefore dismissed. In total, 113 caregiver responses were included in the data analysis.

### Caregivers’ sociodemographic characteristics

The sociodemographic characteristics of the 113 caregivers of pwALS are presented in Table 1. Most caregivers were female (61.9%) with a mean age of 56.4 ± 14.2 years. The majority were either the partner (65.5%) or the offspring (23.9%) of the pwALS. Responses were obtained for all the Portuguese districts, with the majority from the northern region (*n* = 79, 76.1%). Regarding educational attainment, 43.4% had between 9 and 12 years of education, and 36.3% held a degree. In terms of employment status, 49.6% were employed, with a median number of working hours per week of 35 [35; 40], 33.6% were retired and 13.3% were unemployed. Moreover, 33.8% of caregivers reported having stopped working, and 17.6% had reduced their working hours due to caregiving responsibilities.

**Table 1.** Sociodemographic characteristics of caregivers (*n* = 113) of people with amyotrophic lateral sclerosis

	Caregivers (*n* = 113)
**Age, years**	56.4 ± 14.2
**Sex (female), *n* (%)**	70 (61.9%)
**Relationship with the pwALS, *n* (%)**	
Partner	74 (65.5%)
Daughter/Son	27 (23.9%)
Sibling	6 (5.3%)
Parent	5 (4.4%)
Niece/Nephew	1 (0.9%)
**Years of education, *n* (%)**	
≤6 years	23 (20.3%)
9–12 years	49 (43.4%)
Degree	41 (36.3%)
**Working status, *n* (%)**	
Employed	56 (49.6%)
Retired	38 (33.6%)
Unemployed	15 (13.3%)
Sick leave	3 (2.7%)
Student	1 (0.9%)
Total working hours, hours/week	35 [35; 40]
**Stopped working, *n* (%) [*n* = 68]**	
No	33 (48.5%)
Yes	23 (33.8%)
Reduced number of working hours	12 (17.6%)
Household, number of people	2 [2; 3]
Household, number of individuals underage [*n* = 17]	1 [1; 2]
**Household income per month, *n* (%)**	
≤500€	3 (2.7%)
501–1000€	17 (15%)
1001–2000€	46 (40.7%)
>2001€	47 (41.5%)

Data are presented as mean ± standard deviation, median [first quartile; third quartile], or number (percentage), unless stated otherwise.

pwALS, people with amyotrophic lateral sclerosis.

### Financial burden and social benefits

A total of 58 caregivers (51.3%) reported having made structural changes at home, while 19 (16.8%) had not, although such changes were deemed necessary. Additionally, 18 caregivers (15.9%) reported having relocated due to caregiving needs. In terms of social benefits, 25.7% of caregivers reported not receiving any social benefits or support. As for caregivers receiving support, a detailed analysis showed that 75 (66.4%) held “the Medical Certificate of Multi-Purpose Disability,” an official Portuguese document certifying a person’s degree of disability and granting access to social, fiscal, educational, and other related employment benefits. In terms of pecuniary social assistance, 62 (59.9%) reported that their household received the Social Inclusion Benefit (PSI), a monthly support provided to individuals with ≥60% disability, with a maximum value of 316,33€. Additionally, 42 participants (37.2%) received the dependency supplement – an additional component of the PSI awarded to individuals with disabilities living in economic hardship that may increase the benefit by 221,21€ (as of data collection). Regarding formal caregiving support, 11 caregivers (9.7%) employed a personal assistant (formal caregiver) to aid them during specific hours or periods of the day. Other notable entitlements included a parking card for persons with disabilities held by 51 respondents (45.1%), while 4 caregivers (3.4%) had benefited from a respite admission – a service under the National Integrated Continued Care Network that temporarily admits the dependent person to a long-term care unit, thereby providing the caregiver with a break from caregiving duties. Only 16 participants (14.2%) held informal caregiver status – the regulatory framework that acknowledges and governs the rights, duties, and support measures for individuals providing regular or permanent care to dependent persons. Results are summarized in supplementary Table 1.

### Caregiver burden, quality of life, and sleep

Most caregivers (77%) had no prior caregiving experience. The median number of caregiving hours per day was 8 [4; 12]. A majority (66.4%) received help in caregiving tasks, mainly from relatives (69.3%), followed by formal (paid) caregivers or personal assistants (44%), and friends (8%). Results are summarized in Table 2.

**Table 2.** Caregiver burden, quality of life, and sleep and caregivers’ characteristics according to the burden level on ZBI

	All caregivers (*n* = 113)	Low burden (*n* = 39)	High burden (*n* = 73)	*p*-value	Mean difference
**Previously experience as a caregiver?**				0.153	–
Yes	26 (23%)	12 (30.8%)	13 (17.8%)		
No	87 (77%)	27 (69.2%)	60 (82.2%)		
**Hours of caregiving/da**y	8 [4; 12]	6 [3; 10]	8 [4; 14]	0.016	−2.8 (−5.4; −0.1)
**Does anyone help you in the caregiving tasks?**				0.153	–
Yes	75 (66.4%)	12 (30.8%)	13 (17.8%)		
No	38 (33.6%)	27 (69.2%)	60 (82.2%)		
**Who helps?**					–
Relatives	52 (69.3%)	20 (80%)	31 (63.3%)	0.118	
Formal (paid) caregivers or personal assistants	33 (44%)	8 (32%)	24 (49%)	0.217	
Friends	6 (8%)	3 (12%)	3 (6.1%)	0.657	
**Structural changes at home, *n* (%)**					–
Need to relocate, *n* (%)				0.004	
Yes	58 (51.3%)	6 (15.4%)	12 (16.4%)		
No	36 (31.9%)	33 (84.6%)	45 (61.6%)		
No, but it would help	19 (16.8%)	–	16 (21.9%)		
**Characteristics of the person with ALS**					
Age, years	64 [53.5; 72]	59 (52.7%)	66 [57; 73]	0.188	−2.8 (−7.3; 1.7)
Sex (female), *n* (%)	59 [52; 70.5]	23 (59%)	36 (49.3%)	0.427	–
Time with ALS	24 [12; 43.5]	23 [12; 54]	24 [12; 38]	0.816	16.9 (−7.5; 41.3)
**ALSFRS-R**					
Total	21.1 ± 11.31	26.62 ± 11.17	18.15 ± 10.29	<0.001	8.5 (4.3; 12.6)
Mild	10 (8.9%)	7 (17.9%)	3 (4.1%)		
Moderate	34 (30.4%)	18 (46.2%)	16 (21.9%)		
Severe	36 (32.1%)	8 (20.5%)	28 (38.4%)		
Very severe	32 (28.6%)	6 (15.4%)	26 (35.6%)		
Respiratory score	8 [5; 11]	10 [7.5; 12]	7 [4; 10]	0.009	1.8 (0.4; 3.2)
Bulbar function	7 [3; 10]	8 [5; 11]	5 [2; 9]	0.005	2.2 (0.7; 3.7)
Upper limb function	3 [0; 7]	6 [1; 8.5]	2 [0; 6]	0.003	2.4 (1; 3.9)
Lower limb function	2 [0; 6]	4 [1; 7]	1 [0; 5]	0.005	2 (0.6; 3.4)
**Functional level**					
Independent from caregiver	2 (1.8%)	2 (5.1%)	0	0.005	
Slightly dependent on caregiver	5 (4.5%)	4 (10.3%)	1 (1.4%)		
Moderately dependent on caregiver	33 (19.5%)	15 (38.5%)	18 (24.7%)		
Highly dependent on caregiver	32 (28.6%)	10 (25.6%)	22 (30.1%)		
Fully dependent on caregiver	40 (35.7%)	8 (20.5%)	32 (43.8%)		
**Zarit scale**	32 ± 14.8	16.9 (14; 19.8)^a^	40 (38; 42.1)^a^	<0.001	−23.1 (−26.7; −19.5)^a^
No to mild burden	30 (26.8%)	–	–		
Mild to moderate burden	49 (43.8%)	–	–		
Moderate to severe burden	31 (27.7%)	–	–		
Severe burden	2 (1.8%)	–	–		
**SF-36**					
Physical functioning	85 [70; 95]	83.5 (77.1; 90)^a^	76.8 (72.1; 81.5)^a^	0.104	6.7 (−1.4; 14.8)^a^
Role – physical	62.5 [46.9; 87.5]	72.6 (63.8; 81.3)^a^	56.2 (49.8; 62.5)^a^	0.004	16.4 (5.4; 27.4)^a^
Role – emotional	62.5 [41.7; 91.7]	73.3 (64.6; 81.9)^a^	56.9 (50.6; 63.1)^a^	0.003	16.4 (5.6; 27.3)^a^
Vitality	43.8 [31.3; 56.3]	55.3 (49.9; 60.8)^a^	37.6 (33.6; 41.5)^a^	<0.001	17.8 (10.9; 24.6)^a^
Mental health	55 [40; 70]	67.1 (61.1; 73.1)^a^	49.1 (44.8; 53.5)^a^	<0.001	18 (10.4; 25.5)^a^
Social functioning	62.5 [37.5; 87.5]	79.6 (72.2; 87.1)^a^	54 (48.6; 59.4)^a^	<0.001	25.6 (16.3; 34.9)^a^
Bodily pain: experienced pain (past 4 weeks)	60 [40; 80]	2.5 (2.1; 3)^a^	3.2 (2.9; 3.5)^a^	0.016	−0.7 (−1.3; −0.1)^a^
General health	50 [35; 65]	58.7 (53; 64.4)^a^	47 (42.9; 51.1)^a^	0.002	11.7 (4.6; 18.9)^a^
**Sleep related questions**					
Current sleep quality	3 [2; 3]	2.9 (2.6; 3.1)^a^	2.6 (2.4; 2.8)^a^	0.111	0.3 (−0.1; 0.6)^a^
Previous sleep quality	4 [3; 4]	3.4 (3.1; 3.6)^a^	3.7 (3.5; 3.9)^a^	0.032	−0.4 (−0.7; −0.03)^a^
Hours of sleep per night	6 [5; 7]	6 (5.6; 6.5)^a^	6.1 (5.8; 6.4)^a^	0.707	−0.1 (−0.6; 0.4)^a^
Number of awakenings per night	2 [1; 3]	2.1 (1.4; 2.8)^a^	3.1 (2.6; 3.7)^a^	0.023	−1 (−1.9; −0.1)^a^
Are the awakenings related to caring?				0.049	–
Never	35 (31.3%)	16 (41%)	18 (25%)		
Sometimes	32 (28.6%)	14 (35.9%)	18 (25%)		
Most time	23 (20.5%)	4 (10.3%)	19 (26.4%)		
Always	22 (19.6%)	5 (12.8%)	17 (23.6%)		
Number of awakenings related to patient ventilator (*n* = 67)	2 [0; 3]	1.8 (−0.3; 3.9)^a^	3.8 (2.4; 5.2)^a^	0.118	−2 (−4.5; 0.5)^a^
Time to fall back asleep				0.041	–
<15 minutes	18 (23.4%)	12 (41.4%)	12 (18.8%)		
15–30 minutes	36 (46.8%)	12 (41.4%)	29 (45.3%)		
>30 minutes	23 (29.9%)	5 (17.2%)	23 (35.9%)		

Data are presented as mean ± standard deviation, median [first quartile; third quartile], mean difference (95% confidence interval), or number (percentage), unless stated otherwise.

pwALS, people with amyotrophic lateral sclerosis.

a Analysis adjusted for caregivers’ age, sex, socioeconomic status, and previous caregiving experience.

Mean caregiver burden, as measured by the ZBI, was 32 ±  14.8. Most caregivers (43.8%) reported mild to moderate burden, while 27.7% reported higher levels, including 1.8% with severe burden.

Regarding the SF-36, the mean general health score was 50 [35; 65]. The highest scores were observed in physical functioning (85 [70; 95]), social functioning (62.5 [37.5; 87.5]), role functioning/emotional (62.5 [41.7; 91.7]), and role functioning/physical (62.5 [46.9; 87.5]). Lower scores were reported for mental health (55 [40; 70]) and vitality (43.8 [31.3; 56.3]).

Statistically significant higher SF-36 scores (Table 2) were observed in caregivers with low burden compared to those with high burden, particularly in role-physical (*p* = 0.004), role-emotional (*p* = 0.003), vitality (*p* < 0.001), mental health (*p* < 0.001), social functioning (*p* < 0.001), bodily pain (*p* = 0.016), and general health (*p* < 0.001).

Sleep-related outcomes revealed a median current sleep quality score of 3 [2; 3] and a previous sleep quality score of 4 [3; 4], with a significant decrease in sleep quality due to caregiving (*p* < 0.001). Caregivers reported to sleep a median of 6 [5; 7] hours per night. The median number of awakenings per night was 2 [1; 3]. Awakenings were related to caregiving on most or all nights for 40.9% of participants, and among those caring for ventilated patients (*n* = 67), a median 2 [0; 3] awakenings were due to the ventilator.

Caregivers with high burden reported poorer current sleep quality compared to those with low burden (*p* = 0.026) and experienced a higher number of awakenings (*p* = 0.023), and more frequently reported that these awakenings were “most of the time” or “always” related to caregiving (50% vs. 23.1%, *p* = 0.049). They also took longer than 30 minutes to fall back asleep (*p* = 0.041) and reported significantly better previous sleep quality (*p* = 0.029).

Caregivers with high burden were more likely to report that they had to relocate, or that although they had not relocated, it would have been helpful, compared with caregivers with low burden (*p* = 0.004). They also reported spending more hours per day on caregiving (*p* = 0.016), and a positive correlation was observed between ZBI score and hours of caregiving (*r* = 0.274, *p* = 0.003). The general health of pwALS is presented in Table 2.

### Patient characteristics – Caregivers’ views

The characteristics of pwALS are presented in Table 2 and Supplementary Table 2. The mean ALSFRS-R score was 21.1 ± 11.3. Based on ALSFRS-R classifications, 8.9% of participants were categorized as having mild disease, 30.4% moderate disease, 32.1% severe disease, and 28.6% very severe disease.

Regarding levels of dependence, 35.7% of participants were fully dependent on caregivers, 28.6% were highly dependent, 19.5% were moderately dependent, 4.5% were slightly dependent, and 1.8% were fully independent.

The most used assistive device was the ventilator (71.4%), and pwALS cared for by caregivers with high burden were more likely to use ventilators compared with those cared for by caregivers with low burden (*p* = 0.048).

Caregivers experiencing high burden were more likely to care for patients with lower ALSFRS-R scores than those with low burden (*r* = −0.411, *p* < 0.001). Specifically, the high-burden group included more pwALS classified as severe or very severe on the ALSFRS-R scale (*p* < 0.001).

## Discussion

This study provides an overview of the challenges caregivers of pwALS face, highlighting the profound impact of caregiving responsibilities on their personal, professional, and psychological well-being. Caregiver burden is a significant concern for informal caregivers, who are typically family members or individuals with an emotional bond to the patient, and are often unpaid. Consistent with other studies, most caregivers of pwALS in our study are female, often spouses, and are providing care for the first time (Kennedy et al. 2022; Conroy et al. 2023).

A significant proportion of caregivers in our study reported having stopped working or reduced their working hours due to caregiving duties, highlighting the economic challenges that frequently impact informal caregiving. Similar patterns have been observed in caregivers of people with other chronic diseases. For example, in chronic obstructive pulmonary disease, around one third of working-age caregivers reported profession-related problems (Miravitlles et al. 2015). In cancer, extended periods of work leave are not uncommon (de Moor Js et al. 2017), and among caregivers of people with dementia, including Alzheimer’s disease, reduced working hours or unemployment have been linked to poorer physical health compared with working caregivers without such reductions (Socci et al. 2021). Overall, these findings suggest that the professional and health consequences of caregiving, although not exclusive to ALS, represent a common challenge in the daily life of caregivers of pwALS.

Financial difficulties, resulting from loss of income and increasing costs associated with ALS care, can complicate the socioeconomic burden, even in the early stages of the disease (Obermann and Lyon 2015). Notably, a quarter of caregivers reported not receiving any social benefits. This shortfall highlights not only systemic gaps but also the bureaucratic delays and interconnectedness of aid programs, which are often reliant on prior approvals, side by side with an uneasy rare disease diagnosis with different progression rates (Sennfält et al. 2023). The system is therefore ill-equipped to provide prompt support for rapidly changing, uncommon, and rare health and social issues, resulting in fragmented and uncoordinated care pathways. These gaps can impair the ability to care for these populations and should be addressed (Nivakoski and Baggio (2025)). The creation of social support networks could improve caregivers’ and patients’ support in these complex matters, emerging as an area for development to optimize these issues. Evidence suggests that targeted caregiver support policies, including financial subsidies and respite care, are associated with better preservation of caregiver health (Calvó-Perxas et al. 2021), which may, in turn, reduce emergency healthcare utilization and delay patient institutionalization, outcomes linked to substantially higher public healthcare costs. Expanding social support is thus not only a matter of welfare but a strategic development to optimize resource allocation and improve the sustainability of long-term care in ALS.

Home care is the primary approach for managing pwALS, these problems are exacerbated by the frequent need to implement structural changes that ensure accessible living environments at home, in line with the progressive physical deterioration associated with ALS. One third of caregivers reported a need to be relocated to better conditions, and two thirds reported a need for structural changes in the home environment. These, while essential for patient safety and comfort, represent an additional factor impacting financial and psychological status (Schönfelder et al. 2020). In other populations, such as caregivers of patients with dementia, these major household adaptations may not be needed. Generally, only safety adaptations are required as cognitive decline increases (Young et al. 2023). Therefore, even though other chronic disorders might require adaptations, the ones required for pwALS appear to impose a more serious challenge, further worsening the caregivers’ well-being. There is limited research on enhancing care for pwALS by adapting the home environment, especially considering regional disparities observed within countries and between different healthcare and social support structures internationally, which can directly impact the burden of care. For instance, the Netherlands and Germany offer governmental aid programs for home adaptations for ALS patients (van den Berg Jp et al. 2003; Funke et al. 2018), while countries like Canada can provide partial financial refunds for structural modifications by combining federal, provincial, and local sources (Gladman et al. 2014). Neither approach is widely implemented nor readily available in Portugal. These pressing matters could also be addressed through an improved support network and financial and social security support.

Caregivers’ HRQoL reveals an impairment in mental health and vitality parameters, with lower scores, in comparison with physical functioning scores. This disparity suggests that, while caregivers may maintain physical capabilities, their emotional resilience and energy levels are significantly compromised, impacting their mental well-being – a concern that cannot be overlooked and that can aggravate the challenges of caring for pwALS. When comparing our results with normative data for the Portuguese population, caregivers of pwALS reported substantially lower scores in several SF-36 domains. General health perception was worse, and marked differences were observed in social functioning, as well as in role limitations due to both emotional and physical problems. Particularly concerning were the much lower scores in mental health and vitality, reinforcing the psychological and emotional burden associated with caregiving. In contrast, physical functioning was similar to that of the general population, suggesting that the negative impact of caregiving is more pronounced in psychosocial rather than physical dimensions (Ferreira et al. 2012). Further problems might emerge, even after the patients’ death, with studies evidencing an increased mortality for individuals involved in caregiving, reinforcing the need to address caregivers’ HRQoL (Schulz and Beach 1999; Nivakoski and Baggio 2025). These disruptions likely trigger a state of chronic hypervigilance, where the caregiver remains in lighter stages of sleep to maintain alertness for potential emergencies, such as ventilator alarms or signs of respiratory distress (Cichoń et al. 2025). Although this phenomenon has been well documented in parents of ventilator-assisted children, where sleep instability is a known predictor of poorer HRQoL (Meltzer et al. 2015), our findings suggest that ALS caregivers face a comparable burden. The negative impacts of such sleep deprivation may directly undermine caregivers’ capacity to provide safe care and preserve their own physical health, reinforcing concerns previously raised regarding the burden associated with HMV (Radunovic et al. 2017).

These results underscore that, beyond its clinical benefits for patients, the use of home ventilation can also impose a significant burden on caregivers’ rest, which may further compromise their overall well-being. However, further studies are needed to ascertain the impact of HMV on caregiver burden.

The ZBI showed a predominantly mild to moderate overall level of burden. This is somewhat lower than the moderate to severe levels reported in previous studies with caregivers of pwALS (Schreiner et al. 2006; Ipek and Güneş Gencer 2024). Such differences are unlikely to reflect an underestimation of burden, but may instead relate to sample characteristics, cultural factors, or methodological variations (Ipek and Güneş Gencer 2024). Although the literature has suggested that younger caregivers, particularly those under the age of 55 years, tend to experience greater burden, this association was not observed in our study (Qutub et al. 2014).

Caregivers with lower burden scores reported higher SF-36 scores across all domains, with statistically significant differences in several HRQoL domains. These findings reinforce the inverse relationship between caregiver burden and quality of life. Cognitive interventions, such as mindfulness, have emerged as options to decrease caregiver burden (Pagnini et al. 2014). These might be integrated in a support network to improve caregivers’ HRQoL, in addition to potential benefits in the care of pwALS (Pagnini et al. 2014).

Higher caregiver burden was associated with greater patient disability, as measured by the ALSFRS-R, and with the number of hours of care provided, consistent with other studies (de Wit et al. 2018; Galvin et al. 2018; Ipek and Güneş Gencer 2024). Therefore, as the disease progresses, additional support might be required to compensate for these increasing demands. A lack of caregiving experience or training likely aggravates problems in these advanced stages of disease. Studies have shown that implementing training programs can enhance patient and caregiver satisfaction (Sanjuán et al. 2023) and reduce depression, anxiety, and caregiver burden (Jensen et al. 2015; Walter and Pinquart 2020). The use of similar strategies within a support network might help equip caregivers with adequate caring skills for pwALS and ultimately reduce burden.

Our results showed that most caregivers with high burden did not receive help from others in caregiving tasks, highlighting the potential importance of community-based support and caregiving assistance. Even so, the presence of assistance from others is essential not only for managing practical caregiving tasks but also for coping with the emotional and psychological demands associated with caring for pwALS (Galvin et al. 2018). Anticipation and preventive strategies may help caregivers maintain a sense of normality, as autonomy declines. Accepting dependence becomes increasingly complex, especially given the heavy demands placed on caregivers by pwALS, and their efforts to preserve routine and stability in the face of progressive change (Gonçalves et al. 2025). Limited knowledge of the disease trajectory may contribute to lower levels of caregiver anxiety and burden (Tang et al. 2021). This insight can be considered in practical contexts as a potential strategy to engage caregivers more effectively and to tailor the content and timing of information and discussions throughout the disease journey.

Given the cross-sectional design of this study, the observed associations between caregiver burden, sleep quality, and HRQoL cannot be interpreted as causal, nor can the directionality of these relationships be established.

Our study has some limitations that should be considered when interpreting the findings. Firstly, participation depended on caregivers’ collaboration and willingness, given its consecutive nature. Even though the number of individuals who refused to participate was not reported, possible biases, including the exclusion of more burned-out volunteers, cannot be ruled out. Secondly, despite the diffusion of our data collection method via electronic means to reach individual participants nationwide, this recruitment strategy may have introduced a selection bias due to caregivers’ lower digital proficiency. This may have resulted in a sample predominantly composed of caregivers who are more digitally connected or socially engaged through associations, potentially overlooking those in more isolated or lower socioeconomic conditions, which impacts the transferability of the findings. Although we included participants from all the country’s districts, a predominance of participants from the northern area was observed. Additionally, while further analyses adjusted for key caregiver characteristics, including age, sex, socioeconomic status, and previous caregiving experience, residual confounding from unmeasured factors cannot be fully excluded. Finally, the reliance on self-reported data may have introduced recall and social desirability bias, as well as potential misclassification of caregiver characteristics, comorbidities, and sleep outcomes. These limitations should be considered when interpreting the observed associations, particularly with regard to causal or directional inferences.

### Highlights

- Higher caregiver burden in ALS is associated with patient disability and increased caregiving hours.
- Mental health and vitality are more impaired than physical health among ALS caregivers.
- A greater burden is linked to poorer sleep quality in caregivers.
- Early identification of at-risk caregivers should inform targeted interventions.
- One in four caregivers in Portugal reports no access to social benefits.

## Conclusion

The data presented here emphasize the urgent need for comprehensive caregiver support programs. Interventions should address both practical needs (e.g., financial aid, home modifications, access to professional care) and emotional well-being (e.g., counselling, peer support groups). Policymakers must consider the hidden costs borne by informal caregivers and ensure that social benefits are accessible, adequate, and responsive to the evolving demands of ALS care.

Early identification of caregivers at risk of high burden – especially those with no prior experience – could enable proactive support and potentially mitigate long-term negative outcomes. Health professionals should routinely assess caregiver well-being as part of ALS management, recognizing that caregiver health directly influences patient outcomes.

## Supporting information

10.1017/S1478951526101825.sm001 Gonçalves et al. supplementary material Gonçalves et al. supplementary material

## Supplementary material

The supplementary material for this article can be found at https://doi.org/10.1017/S1478951526101825.
