# Supplementary material for: The burden of care: Health and wellbeing of informal caregivers of people with amyotrophic lateral sclerosis
Source: Palliat Support Care. 2026 Feb 25;24:e65. doi: 10.1017/S1478951526101825 (PMC13166376; doi:10.1017/S1478951526101825)
Supplement: Gonçalves et al. supplementary material [file S1478951526101825sup001.docx]

*Supplementary table 1* *– Financial burden and social benefits, according to caregivers of pwALS (n=113).*

| Structural changes at home, n(%)  Yes  No  No, although they are necessary | 58 (51.3%)  36 (31.9%)  19 (16.8%) | Observation  Modifications to the home environment to accommodate caregiving needs |
| --- | --- | --- |
| Need to relocate, n(%)  Yes  No  No, but it would help | 18 (15.9%)  79 (69.9%)  16 (14.2%) | to better meet the dependent person’s care requirements |
| Social benefits, n (%)  Medical Certificate of Multi-Purpose Disability  Disabled parking card  Social inclusion benefit (PSI)  Dependency supplement  Informal caregiver status  With the main informal caregiver status  Personal assistant  Informal caregivers' rest  None | 75 (66.4%)  51 (45.1%)  20 (17.7%)  42 (37.2%)  16 (14.2%)  3 (2.7%)  11 (9.7%)  4 (3.5%)  29 (25.7%) | Official Portuguese certificate of a person’s degree of disability and granting access to social, fiscal, educational, and employment benefits  A card granting parking privileges for persons with disabilities  Monthly financial support for individuals with ≥ 60% disability, up to 313,31€  Additional component of the PSI for individuals with disabilities in economic hardship, increasing the benefit by €221.21€  Regulatory framework recognising and governing rights, duties, and support measures for individuals providing regular or permanent care to dependents  Formal assistant, provided through a publicly funded application program, hired to support a patient during designated periods of the day in daily and social tasks that foster independence  Service within the National Integrated Continued Care Network that temporarily admits the dependent person to a care unit for caregiver respite |
| Costs, euros/month  Conventional Therapies [n=100]  Medication [n=112]  Transportation to therapy sessions [n=83]  Nutritional supplements [n=100]  Incontinence and hygiene care materials [n=112]  Non-nutritional supplements [n=78]  Complementary therapies [n=72] | 72 [2; 115]  50 [30; 100]  40 [0; 65]  40 [0; 60]  10 [0; 50]  0 [0; 2]  0 [0; 0] | Rehabilitation, respiratory therapy, Speech-Language Therapy, and others.  Prescribed by a registered dietitian–nutritionist  Often promoted by alternative therapies, used despite partial or total lack of clinical evidence |

Data are presented as median [first quartile; third quartile] or number (percentage), unless stated otherwise.

pwALS, people with amyotrophic lateral sclerosis.

*Supplementary Table 2* *– Supplementary Table 2 – Patient support devices and corresponding levels of dependence in daily care tasks.*

| Support devices  Ventilator  Backup ventilator  Mechanical insufflator-exsufflator  Transit wheelchair  Comfort wheelchair  Electric wheelchair  Shower/toilet chair  Patient transfer hoist  Adjustable hospital bed  Alternative communication systems  Adapted call bell/alarm system  Comfort armchair/sofa (electric)  Wheelchair-adapted van/car  Toilet seat riser | 80 (71.4%)  27 (24.1%)  55 (49.1%)  64 (57.1%)  32 (28.6%)  27 (24.1%)  69 (61.6%)  18 (16.1%)  59 (52.7%)  39 (34.8%)  29 (25.9%)  42 (37.5%)  7 (6.3%)  23 (20.5%) |
| --- | --- |
| Levels of dependence |  |
| Assist in wearing a mask [n=79] |  |
| No need of help  Only supervision  Help during the task  Full help | 16 (20.3%)  8 (10.1%)  10 (12.7%)  45 (56.9%) |
| Assist in removing a mask [n=79] |  |
| No need of help  Only supervision  Help during the task  Full help | 19 (24.0%)  7 (8.9%)  14 (17.7%)  39 (49.4%) |
| Assist in using an In-exsufflator [n=55] |  |
| No need of help  Only supervision  Help during the task  Full help | 11 (20%)  8 (14.5%)  9 (16.4%)  27 (49.1%) |
| Assist in urination |  |
| No need of help  Only supervision  Help during the task  Full help | 38 (33.9%)  20 (17.9%)  19 (17%)  35 (31.3%) |
| Assist in defecation |  |
| No need of help  Only supervision  Help during the task  Full help | 37 (33%)  18 (16.1%)  22 (19.6%)  35 (31.3%) |
| Assist in food preparation |  |
| No need of help  Only supervision  Help during the task  Full help | 10 (8.9%)  6 (5.4%)  18 (16.1%)  78 (69.6%) |
| Assist in food intake |  |
| No need of help  Only supervision  Help during the task  Full help | 23 (20.5%)  21 (18.8%)  15 (13.4%)  53 (47.3%) |
| Assist in fluid intake |  |
| No need of help  Only supervision  Help during the task  Full help | 29 (25.9%)  22 (45.5%)  9 (8%)  52 (46.4%) |
| Assist with bed mobility |  |
| No need of help  Only supervision  Help during the task  Full help | 21 (18.8%)  16 (14.3%)  18 (16.1%)  57 (50.9%) |
| Assist in transfer/sit to stand |  |
| No need of help  Only supervision  Help during the task  Full help | 22 (19.6%)  18 (16.1%)  15 (13.4%)  57 (50.9%) |
| Assist in the use of electronic devices |  |
| No need of help  Only supervision  Help during the task  Full help | 43 (38.4%)  19 (17%)  19 (17%)  31 (27.7%) |
| Assist in personal hygiene/bathing |  |
| No need of help  Only supervision  Help during the task  Full help | 17 (15.2%)  14 (12.5%)  21 (18.8%)  60 (53.6%) |
| Assist in dressing and undressing |  |
| No need of help  Only supervision  Help during the task  Full help | 15 (13.4%)  7 (6.3%)  27 (24.1%)  63 (56.3%) |
| Assist in mobility |  |
| No need of help  Only supervision  Help during the task  Full help | 19 (17%)  23 (20.5%)  15 (13.4%)  55 (49.1%) |
| Assist in leaving the house |  |
| No need of help  Only supervision  Help during the task  Full help | 12 (10.7%)  13 (11.6%)  20 (17.9%)  67 (59.8%) |

Data are presented as mean ± standard deviation, median [first quartile; third quartile] or number (percentage), unless stated otherwise.
